# The specific heat of the human body is lower than previously believed: The journal *Temperature* toolbox

**DOI:** 10.1080/23328940.2022.2088034

**Published:** 2022-07-22

**Authors:** Xiaojiang Xu, Timothy P. Rioux, Michael P. Castellani

**Affiliations:** a US Army Research Institute of Environmental Medicine, Thermal and Mountain Medicine Division, Natick, MA, USA; b Oak Ridge Institute for Science and Education

**Keywords:** Mean body temperature, heat content, heat storage, calorimetry, thermometry

## Abstract

The specific heat capacity of the human body is an important value for heat balance analysis in thermoregulation and metabolism research. The widely used value of 3.47 kJ · kg^−1^· °C^−1^ was originally based on assumptions and was not measured or calculated. The purpose of this paper is to calculate the specific heat of the body, defined as the mass-weighted mean of the tissue specific heat. The masses of 24 body tissue types were derived from high-resolution magnetic resonance images of four virtual human models. The specific heat values of each tissue type were obtained from the published tissue thermal property databases. The specific heat of the entire body was calculated to be approximately 2.98 kJ · kg^−1^ · °C^−1^ and ranged from 2.44 to 3.39 kJ · kg^−1^ · °C^−1^ depending on whether min or max measured tissue values were used for the calculation. To our knowledge, this is the first time specific heat of the body has been calculated from the measured values of individual tissues. The contribution of the muscle to the specific heat of the body is approximately 47%, and the contribution of the fat and skin is approximately 24%. We believe this new information will improve the accuracy of calculations related to human heat balance in future studies of exercise, thermal stress, and related areas.

## Introduction

The human body generally has the ability to stabilize its internal temperature across a range of ambient temperature values, adjusting physiological mechanisms that maintain heat balance between metabolic heat production and heat loss to the environment. In studies of human thermoregulatory physiology, the derivation of the change in body heat content is of fundamental importance to the assessment of thermal responses and status [1–3]. An important number associated with the heat content is the average specific heat of the human body. Specific heat is the amount of heat per unit mass required to raise the temperature by 1°C. The specific heat value of 3.47 kJ · kg^−1^ · °C^−1^ has been widely used in heat balance calculations. However, this value is originally based on assumptions [2] and was never measured or calculated.

It is a challenge to measure the specific heat of the body, as the body consists of multiple tissue types and the tissue temperatures are inhomogeneous. Numerous studies have measured or estimated the thermal properties of individual tissues or organs [4,5]. The Foundation for Research on Information Technologies in Society (IT’IS) (https://itis.swiss/) recently compiled the thermal property data in the literature, analyzed these data, and created a database for thermal properties of biological tissues [5]. This database included the specific heat values of ~100 biological tissue types. The specific heat of tissue varies significantly, ranging from ~0.7 kJ · kg^−1^ · °C^−1^ for tooth (enamel) to 4.2 kJ · kg^−1^ · °C^−1^ for eye (sclera).

Masses of 24 body tissue types, which were derived from high-resolution medical images, are available [6,7]. Thus, the data required to determine the specific heat of the body are available and can be used for calculation. The purpose of the present analysis was, for the first time, to quantify the average specific heat of the total human body using individual tissue values for mass and specific heat.

## Methods

The specific heat of the human body is the average specific heat of tissues with respect to the masses, and thus is calculated from the specific heat of each tissue and its mass by the following equation:

(1) $$C_{p}=\overset{n}{\underset{i=1}{\sum }}\frac{m_{i}\cdot C_{p,i}}{m}$$

where C_p_ is the specific heat of the body in kJ · kg^−1^ · °C^−1^, i is the tissue index, n is the total number of tissue types, m_i_ is the tissue mass, and m is the total body mass.

Twenty-four tissue types of four human bodies were used for calculation [7]. These tissue masses were derived from four human models of the Virtual Family [6]. The Virtual Family consists of human models created from high-resolution magnetic resonance imaging of healthy Caucasian European volunteers, and the virtual “individuals” are shown in Table 1. The Virtual Family datasets are composed primarily of 1 mm voxels, representing about 80 tissue types. The datasets were further processed to obtain the masses of 24 tissue types [7]. Table 2 shows the tissue masses of these four volunteers.

**Table 1.** Characteristics of anatomical models.

Data pseudonym	Age (years)	Sex	Height (m)	Mass (kg)
Duke	34	Male	1.74	70
Ella	26	Female	1.60	58
Billie	11	Female	1.46	36
Thelonious	6	Male	1.17	20

**Table 2.** Tissue masses in g (kg · 10^−3^) and specific heat of each tissue in kJ · kg^−1^ · °C^−1^.

	Mass (g)				Specific heat (kJ · kg^−1^ · °C^−1^)		
Tissue/Organ	Duke	Ella	Billie	Thelonius	Average	Min	Max
Adipose	11,830	14,256	5363	3605	2.348	1.806	2.973
Adrenals	11	13	9	3	3.513	3.425	3.600
Brain	1373	1324	1248	1313	3.630	3.578	3.682
Colon Wall	535	338	275	225	3.595	3.595	3.595
Eye Lens	0.30	0.33	0.26	0.32	3.133	3.000	3.664
Gall Bladder	19	25	20	8	3.716	3.716	3.716
Heart	752	599	278	257	3.652	3.457	3.812
Kidney	359	269	165	137	3.763	3.653	3.891
Liver	1244	866	871	586	3.540	3.332	3.617
Lungs	2521	1714	900	595	3.886	3.886	3.886
Muscle	33,842	22,291	14,282	6289	3.421	2.624	3.799
Esophagus	56	29	9	11	3.500	3.500	3.500
Pancreas	70	7	25	11	3.164	2.822	3.506
Red Bone Marrow	959	302	333	111	2.666	2.666	2.666
Skeleton	7857	5987	5259	2541	1.313	0.826	1.650
Skin	5491	3532	2758	1490	3.391	3.150	3.662
Small Intestine	631	624	405	59	3.595	3.595	3.595
Spleen	147	172	148	129	3.596	3.376	3.724
Testes	18	0	0	3	3.778	3.778	3.778
Teeth	31	23	32	12	1.255	1.255	1.255
Thymus	4	20	30	30	3.043	3.043	3.043
Thyroid	9	13	11	0	3.609	3.609	3.609
Breast	0	583	0	0	2.960	2.960	2.960
Uterus	0	52	20	0	3.676	3.676	3.676
Blood	2438	4261	1559	1185	3.617	3.300	3.900

The thermal properties of body tissue have been measured or estimated in over 150 studies [4], and these values have been evaluated, analyzed, and compiled in two databases [4,5]. The ITIS database includes specific heat values for about 100 tissue types [5] and are used in this calculation. Table 2 shows the specific heat values of 24 tissues. For each tissue, the specific heat includes the average value, and the minimum and maximum of source values.

Tissue mass from [7] and tissue specific heat from [4,5].

## Results

We calculated the specific heat of the body from the mass and specific heat of tissues in Table 2 using Equation (1), and repeated the calculation three times for each virtual model using the specific heat values in Table 2: the average, min, and max values. The results are shown in Table 3.

**Table 3.** Average specific heat of the body in kJ · kg^−1^ · °C^−1^.

Data pseudonym	Average	Min	Max
Duke	3.028	2.460	3.389
Ella	2.965	2.423	3.346
Billie	2.954	2.413	3.305
Thelonious	2.972	2.476	3.317
Mean	2.980	2.443	3.339

## Discussion

The major new finding of the present analysis is that the specific heat of the body was approximately 2.98 kJ · kg^−1^ · °C^−1^, and ranged from 2.44 to 3.34 kJ · kg^−1^ · °C^−1^ depending on whether min or max measured tissue values were used for the calculation. The value of 2.98 kJ · kg^−1^ · °C^−1^ is approximately 17% lower than the widely used and assumed values of 3.47 kJ · kg^−1^ · °C^−1^ [2]. To the best of our knowledge, this is the first time values for body specific heat have been directly calculated according to the definition in Equation (1), using the most up-to-date specific heat values of different tissue types.

Our method is novel and calculates the specific heat of the body from the tissue masses and tissue specific heat according to the definition of the body specific heat described in Equation (1). A calorimeter can be used to measure the specific heat of individual tissue [4], but not the specific heat of the whole body. This is primarily because the calculation of the mean body temperature, either from the core and skin or from the core, muscle, and skin temperatures, requires coefficients that are inconsistent across studies, difficult to determine accurately, and dependent on the body thermal status [1,8,9]. In our new method, the masses of body tissues are derived from the medical images of volunteers [6,7]. Many specific heat values in Table 2 were measured and some were estimated from the tissue composition and water content [4,5]. Thus, the specific heat determined by our new method can be considered a measured value. As heat content is an important parameter for the assessment of human thermal status [1,10–16], we believe this new information will improve the accuracy of calculations related to human heat balance in future studies of exercise, thermal stress, and related areas.

The muscle contribution to the specific heat of the body is approximately 47%; fat and skin contribute approximately 24%, and each of the remaining tissue types contribute 7% or less. Thus, the specific heat of any individual human body is directly related to body composition. For example, a study in mice demonstrated that the specific heat varied with body fat percentage. The specific heat was 2.65 kJ · kg^−1^ · °C^−1^ for obese mice with 53% fat and 3.66 kJ · kg^−1^ · °C^−1^ for lean mice with 8% fat [17]. It was proposed that the specific heat of the human body was also adjusted according to fat percentage [18,19]. This indicates that the body heat content, an important parameter in thermoregulation, is related to body composition as well. Thus, when calculating the body heat content, a two-compartment thermometry model (core and shell) predicted the heat content more accurately than one-compartment thermometry model [19] and a three-compartment thermometry model (core, muscle, and shell) predicted the heat content more accurately than a two-compartment thermometry model [1,8,20]. Since skeletal muscle contributes almost 50% of the body specific heat, this tissue should be specifically considered in the calculation and analysis of the body heat content or mean body temperature.

Given the fact that the value 3.47 kJ · kg^−1^ · °C^−1^ has been widely used, it is necessary to analyze the differences between this value and our new values. Burton’s paper did not provide tissue masses required for the calculation of mass-weighted specific heat but mentioned that the specific heats of tissues vary from 2.93 kJ · kg^−1^ · °C^−1^ for fat to a value close to 4.18 kJ · kg^−1^ · °C^−1^ for blood [2]. If the blood specific is assumed to be 3.97 kJ · kg^−1^ · °C^−1^ (nearly unity 0.95 kcal · kg^−1^ · °C^−1^), then the average of the fat and blood specific heats is exactly 3.47 kJ · kg^−1^ · °C^−1^. In other words, Burton’s estimation was based on the assumption that a human body consists of 50% fat and 50% blood. The specific heats of fat and blood in Burton’s paper are only slightly higher than the maximum values in Table 2. Therefore, inaccurate tissue masses are likely the main reasons for discrepancies between the value 3.47 kJ · kg^−1^ · °C^−1^ and our values.

Tissue masses determined from medical image databases make it possible to calculate the specific heat of the body. In fact, medical image datasets have been used to develop local and whole-body thermoregulation models [21–26]. Medical image datasets were also used to determine skin thinness and compartment/layer sizes for thermoregulation models [7,27]. Therefore, medical imaging provides an opportunity and necessary data to improve and enhance research of temperature regulation.

One limiting factor in the present study is that we included only the four virtual individuals – two adults and two children. The inter-individual difference in the specific heat seems to be small, less than 2%, as shown in Table 3. Our goal in future work is to expand the present analyses to include a larger population when additional data are available. This will allow the examination of the relationship between the body specific heat and individual characteristics, such as fat percentage and lean mass.

## Conclusions

In the present study, we calculated the specific heat of the body according to its definition using masses and published specific heat values of individual tissue types. We report here that the specific heat of the entire body is approximately 2.98 kJ · kg^−1^ · °C^−1^, with a range of 2.44 to 3.34 kJ · kg^−1^ · °C^−1^ depending on whether min or max measured tissue values were used for the calculation. The specific heat value calculated in this study is 17% lower than the widely used value. The contribution of the muscle to the specific heat of the body is approximately 47%, and the contribution of the fat and skin is approximately 24%.

## References

[1] Kenny GP, Jay O. Thermometry, calorimetry, and mean body temperature during heat stress. Compr Physiol. 2013;3(4):1689–1719. 24265242 10.1002/cphy.c130011

[2] Burton AC. Human calorimetry: the average temperature of the tissues of the body. J Nutr. 1935;9(3):261–280.

[3] Kingma BRM, Frijns AJH, and Schellen L, et al. Beyond the classic thermoneutral zone. Temperature. 2014;1(2):142–149. doi:10.4161/temp.29702. PMC4977175 27583296

[4] Mcintosh RL, Anderson VITA. A comprehensive tissue properties database provided for the thermal assessment of a human at rest. Biophys Rev Lett. 2010;05(3):129–151.

[5] Hasgall P, Di Gennario F, Baumgartner C, et al. ITIS database for thermal and electromagnetic parameters of biological tissues. Version. 2022 Feb 22;4:1.

[6] Christ A, Kainz W, Hahn EG, et al. The virtual family–development of surface-based anatomical models of two adults and two children for dosimetric simulations. Phys Med Biol. 2010;55(2):N23–N38. 20019402 10.1088/0031-9155/55/2/N01

[7] Xu X, Rioux TP, MacLeod T, et al. Measured body composition and geometrical data of four “virtual family” members for thermoregulatory modeling. Int J Biometeorol. 2017;61(3):477. 27543100 10.1007/s00484-016-1227-7

[8] Jay O, Gariepy LM, Reardon FD, et al. A three-compartment thermometry model for the improved estimation of changes in body heat content. Am J Physiol Regul Integr Comp Physiol. 2007;292(1):R167–R175. 16931653 10.1152/ajpregu.00338.2006

[9] Aoyagi Y, McLellan TM, Shephard RJ. Determination of body heat storage: how to select the weighting of rectal and skin temperatures for clothed subjects. Int Arch Occup Environ Health. 1996;68(5):325–336. 8832298 10.1007/BF00409418

[10] Brajkovic D, Ducharme MB, Frim J. Relationship between body heat content and finger temperature during cold exposure. J Appl Physiol. 2001;90(6):2445–2452. 11356812 10.1152/jappl.2001.90.6.2445

[11] Jay O, Kenny GP. The determination of changes in body heat content during exercise using calorimetry and thermometry. J Human Environ Syst. 2007;10(1):19–29.

[12] Snellen JW. An improved estimation of mean body temperature using combined direct calorimetry and thermometry. Eur J Appl Physiol. 2000;82(3):188–196. 10929212 10.1007/s004210050671

[13] Looney DP, Long ET, and Potter AW, et al. Divers risk accelerated fatigue and core temperature rise during fully-immersed exercise in warmer water temperature extremes. Temperature. 2019;6(2):150–157. doi:10.1080/23328940.2019.1599182. PMC6620004 31312674

[14] Vallerand AL, Savourey G, Hanniquet AM, et al. How should body heat storage be determined in humans: by thermometry or calorimetry? Eur J Appl Physiol Occup Physiol. 1992;65(3):286–294. 1396660 10.1007/BF00705095

[15] Sawka MN, Castellani JW. How hot is the human body? J Appl Physiol. 2007;103(2):419–420. 17556501 10.1152/japplphysiol.00592.2007

[16] Riera F, Horr R, Xu X, et al. Thermal and metabolic responses of military divers during a 6-hour static dive in cold water. Aviat Space Environ Med. 2014;85(5):509–517. 24834564 10.3357/asem.3077.2014

[17] Faber P, Garby L. Fat content affects heat capacity: a study in mice. Acta Physiol Scand. 1995;153(2):185–187. 7778459 10.1111/j.1748-1716.1995.tb09850.x

[18] Webb P. Heat storage and body temperature during cooling and rewarming. Eur J Appl Physiol Occup Physiol. 1993;66(1):18–24. 8425508 10.1007/BF00863394

[19] Kakitsuba N, Mekjavic IB. Determining the rate of body heat storage by incorporating body composition. Aviat Space Environ Med. 1987;58(4):301–307. 3579815

[20] Tikuisis P. Heat balance precedes stabilization of body temperatures during cold water immersion. J Appl Physiol. 2003;95(1):89–96. 12639852 10.1152/japplphysiol.01195.2002

[21] Kanawaku Y, Kanetake J, Komiya A, et al. Computer simulation for postmortem cooling processes in the outer ear. Legal Med. 2007;9(2):55–62. 17157049 10.1016/j.legalmed.2006.09.006

[22] Collins CM, Smith MB, Turner R. Model of local temperature changes in brain upon functional activation. J Appl Physiol. 2004;97(6):2051–2055. 15322067 10.1152/japplphysiol.00626.2004

[23] Silva ABCG, Laszczyk J, Wrobel LC, et al. A thermoregulation model for hypothermic treatment of neonates. Med Eng Phys. 2016;38(9):988–998. 27378702 10.1016/j.medengphy.2016.06.018

[24] Castellani MP, Rioux TP, Castellani J, et al. A geometrically accurate 3 dimensional model of human thermoregulation for transient cold and hot environments. Comput Biol Med. 2021;138:104892. 34628207 10.1016/j.compbiomed.2021.104892

[25] Dennis BH, Eberhart RC, Dulikravich GS, et al. Finite-element simulation of cooling of realistic 3-D human head and neck. J Biomech Eng. 2003;125(6):832–840. 14986408 10.1115/1.1634991

[26] Zhang M, Li R, Li J, et al. A 3D multi-segment thermoregulation model of the hand with realistic anatomy: development, validation, and parametric analysis. Build Environ. 2021;201:107964.

[27] Xu X, Tikuisis P. Thermoregulatory modeling for cold stress. Compr Physiol. 2014;4(3):1057–1081. 24944030 10.1002/cphy.c130047

